# European Society of Organ Transplantation (ESOT) Consensus Statement on Machine Perfusion in Cardiothoracic Transplant

**DOI:** 10.3389/ti.2024.13112

**Published:** 2024-11-22

**Authors:** Cristiano Amarelli, Irene Bello, Clemens Aigner, Marius Berman, Massimo Boffini, Stephen Clark, Marita Dalvindt, Julien de Wolf, Stephan Ensminger, David Gomez de Antonio, Lucas Hoyos, Lucrezia Palmieri, Martin Schweiger, Sandro Sponga, Bettina Wiegmann, Arne Neyrinck, the ESOT Guidelines Taskforce

**Affiliations:** ^1^ Department of Cardiac Surgery and Transplants, Monaldi, Azienda dei Colli, Naples, Italy; ^2^ Institut Clínic Respiratorio, Hospital Clinic of Barcelona, Barcelona, Spain; ^3^ Department of Thoracic Surgery, Medical University of Vienna, Vienna, Austria; ^4^ Transplant Unit, Royal Papworth Hospital, NHS Foundation Trust, Cambridge, United Kingdom; ^5^ Cardiac Surgery Division, Surgical Sciences Department, Citta della Salute e della Scienza, University of Torino, Turin, Italy; ^6^ Department Cardiothoracic Transplant, Newcastle University, Newcastle upon Tyne, United Kingdom; ^7^ Department of Cardiothoracic Surgery, Lund University, Lund, Sweden; ^8^ Department of Thoracic Surgery, Lung Heart Institute, University Hospital of Lille, Lille, France; ^9^ Department of Cardiac and Thoracic Vascular Surgery, University Heart Center Lübeck, Lübeck, Germany; ^10^ Department of Thoracic Surgery, Puerta de Hierro University Hospital Majadahonda, Madrid, Spain; ^11^ Thoracic Surgery, University Hospital Zurich, Zurich, Switzerland; ^12^ Department of Translational Medical Sciences, Monaldi Hospital, University of Campania “Luigi Vanvitelli“, Naples, Italy; ^13^ Department of Congenital Cardiovascular Surgery, Pediatric Heart Center, University Children’s Hospital Zurich, Zurich, Switzerland; ^14^ Division of Cardiac Surgery, Cardiothoracic Department, University Hospital of Udine, Udine, Italy; ^15^ Department of Cardiothoracic, Transplantation and Vascular Surgery, Hannover Medical School, Hanover, Germany; ^16^ Department of Cardiovascular Sciences, Anesthesiology and Algology, KU Leuven, Leuven, Belgium

**Keywords:** machine perfusion, ex-situ heart perfusion, ex-situ lung perfusion, graft preservation, cardio-thoracic transplantation

## Abstract

The machine perfusion (MP) of transplantable grafts has emerged as an upcoming field in Cardiothoracic (CT) transplantation during the last decade. This technology carries the potential to assess, preserve, and even recondition thoracic grafts before transplantation, so it is a possible game-changer in the field. This technology field has reached a critical turning point, with a growing number of publications coming predominantly from a few leading institutions, but still need solid scientific evidence. Due to the increasing need to expand the donor pool, especially in Europe, where the donor age is steeply increased, a consensus has been established to address the growing need and knowledge of machine perfusion in cardiothoracic transplantation, targeting the unmet scientific need in this growing field but also, priorities for development, and regional differences in utilization rates and organizational issues. To address MP in CT, the European Society of Organ Transplantation (ESOT) convened a dedicated Working group comprised of experts in CT to review literature about MP to develop guidelines that were subsequently discussed and voted on during the Consensus Conference that took place in person in Prague during the TLJ 3.0 in November 2022. The findings and recommendations of the Cardiothoracic Working Group on MP are presented in this article.

## Introduction

Heart and lung transplantation are the most commonly used therapies for patients with end-stage lung and heart failures.

In 2019, a record number of more than 4,500 lung transplant procedures were performed at over 260 lung transplant centers worldwide, thanks to clinical and scientific advancements, new types of donations like donation after cardiac deceased controlled and uncontrolled or *Ex-vivo* Lung Perfusion (EVLP) technique [1].

EVLP allows the assessment, reconditioning before transplantation and the use of grafts that would have discharged.

Heart transplantation (HT) is the most commonly used therapy for patients with end-stage heart failure. Despite over 20,000 patients in the United States being eligible for HT each year, only a small percentage of them actually undergo transplantation. Additionally, donor heart non-utilization rates in the United States are high, with an estimated 60%–65% of viable hearts being discarded, further limiting the impact of HT [2]. The low donor heart acceptance rate may be due to the expectation that using marginal donors will result in poor outcomes.

Preservation of thoracic grafts is crucial to maintain their function during storage. The mainstream method of organ preservation during the last 40 years has been hypothermic preservation by static cold storage (SCS). However, the extension of donor ages has led to the use of grafts that are more vulnerable to ischemic damage. This epidemiologic change has prompted the need for new technologies to recondition the organs and expand the acceptability criteria for heart donation [3].

*Ex-situ* machine perfusion (MP), or *ex-vivo*, is an emerging technique to preserve solid organs explanted for allogeneic organ transplantation. MP provides a more “physiologic” alternative to the standard of care static-cold preservation, allowing for prolonged preservation and real-time monitoring of organ quality. It can also reduce or prevent ischemia-reperfusion injury and potentially convert the time of transport into a potential benefit for the organ, during which the organ can be reconditioned or even healed. Moreover, it has enabled the expansion of donor criteria, including after circulatory death, thereby increasing the organ pool. The MP platform has the potential to be a game-changer by providing reconditioning, modification of diseased organs, and regenerative approaches [4].

In recent years, due to changes in allocation policies and the complicated clinical and surgical profile of cardiac and lung recipients, graft preservation in organ transplantation has once again become a research priority. Improvements in the medical management of outpatients suffering from chronic heart failure and the availability of left ventricular assist devices (LVADs) and ECMO have shifted the allocation of organs to urgent candidates. However, this has led to an increase in ischemic times and an increased chance of primary graft dysfunction (PGD) due to the rise of surgical complexity and the addition of donor and recipient risk factors [5, 6].

The issue of organ preservation in heart transplantation has been flawed by assessing donor quality and possible modifications due to brain death and its management. The graft function after 24–48 h from reperfusion is quite worse than that seen during the evaluation of the graft during retrieval [7]. Within these changes, there are several factors to consider, such as the intrinsic quality and function of the graft during retrieval, the amount of ischemic damage, the amount of damage due to freezing, rewarming, and reoxygenating injury, and the amount of reperfusion injury, which could be related to ischemia and immunologic reasons.

PGD has a dreadful course, affects postoperative ICU stays, and may require expensive treatments like ECMO and temporary circulatory support, affecting ICU stay, costs, morbidity, and mortality. Therefore, alternative sustainable paradigms to improve CT organ preservation are being researched.

Despite initial encouraging data, preservation technologies still await a breakthrough. Optimal assessment parameters are required to evaluate organ quality and viability and must be agreed on.

There is a solid unmet scientific need for well-designed trials or granular data to ascertain the real benefit of MP in each specific subset of donors and recipients. This consensus report was considered timely to define the role of Cardiothoracic machine perfusion and the level of evidence supporting their use in everyday clinical practice. Furthermore, these data are required to support decision-making, pharmacoeconomic evaluations, and logistical and organizational models that may be sustainable in different social and healthcare systems. Moreover, MP could provide:

• An organizational paradigm shift to increase the number of transplants.
• Providing opportunities for assessment.
• Drug therapies.
• Cellular therapies.
• Facilitating further research and innovation.

Aim of the guidelines: To address Machine perfusion in cardiothoracic transplant, ESOT convened a consensus conference comprised of a global panel of experts involving six transplant experts for the heart and six for the lung to develop expert opinion on key aspects of MP in CT transplant and to help define future needs for research. Summaries of the evidence were presented to the entire group of panelists and jury (MB). The consensus findings and recommendations of the ESOT Consensus guidelines on MP are presented in this document. This document, which will be updated to reflect new evidence as it becomes available, is intended for healthcare providers.

## Methods

A dedicated Guidelines Taskforce within ESOT organized the consensus development process and its sections ELITA, EKITA, EPITA, ECTTA, ETHAP, Education Committee, YPT, Transplant International editorial board members, and patient representatives. A detailed description of the methodology used has been reported previously [8].

Briefly, key issues related to MP in CT transplant topics were identified by each working group, and specific clinical questions were formulated according to the PICO methodology (PICO = Population, Intervention, Comparator, and Outcome) [9]. All PICO questions are listed in Table 1. Following the definition of the PICOs, literature searches were developed by expert staff from the CET (Center of Evidence in Transplantation) who have expertise in conducting systematic reviews and subsequently integrated, when needed, by the steering committee experts.

**TABLE 1:** Heart and Lung Pico’s proposed to CET.

Heart	
PICO 1: Heart	In heart transplantation, for which heart should machine perfusion be performed?
PICO 2: Heart	Heart In heart transplantation, which protocol/perfusate/perfusion strategy for ex-vivo/ex-situ heart perfusion leads to the best clinical outcomes post-transplant?
PICO 3: Heart	In heart transplantation, which biomarker/parameter is capable to predict the graft survival, graft function, primary non-function during ex vivo heart perfusion?
PICO 4: Heart	In heart transplantation, which recipients will benefit from a heart assessed by machine perfusion?
Lung	
PICO 1: Lung	In lung transplantation, for which type of lung should ex vivo lung perfusion be performed?
PICO 2: Lung	In lung transplantation, which protocol/perfusate/ventilation strategy for ex-vivo/ex-situ lung perfusion leads to optimal outcomes?
PICO3: Lung	In lung transplantation, which parameters (physiological, biomarkers) should be used to determine graft quality during ex vivo lung perfusion?
PICO4: Lung	In lung transplantation, which recipients should benefit from a lung assessed by ex vivo lung perfusion?

**FIGURE 1:**
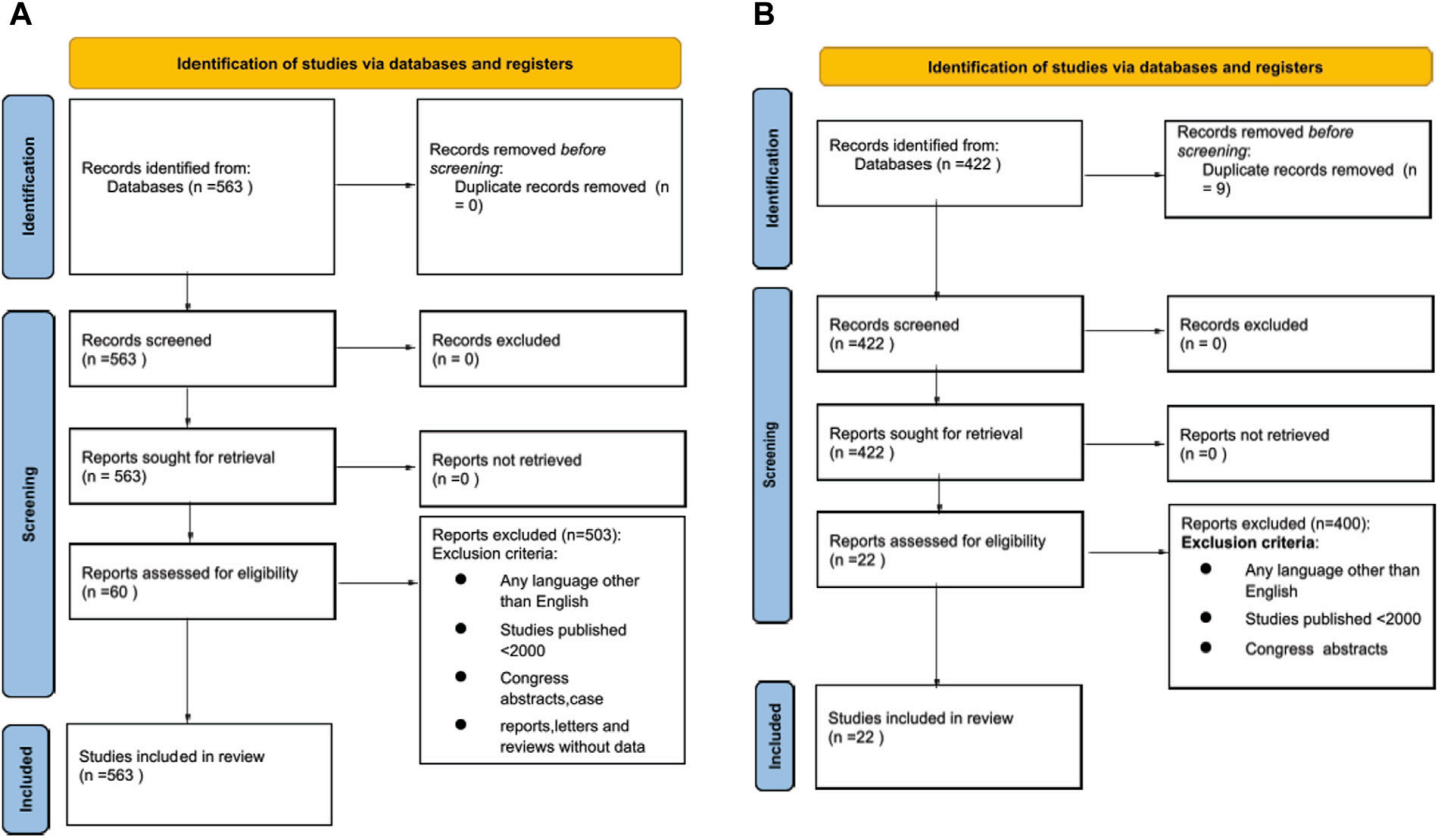
**(A)** Prisma Flow Chart of literature search for Heart Machine Perfusion. **(B)** Prisma Flow Chart of literature search for Lung Machine Perfusion.

A PRISMA flowchart describing the number of studies identified by the literature search and the number of studies selected for inclusion in the consensus statement appears in Figures 1A, B.

A summary of the evidence addressing each key question by the included studies was prepared in evidence (Supplementary Tables S2, S3). The workgroup proposed a recommendation for each key question based on the quality of evidence rated using the GRADE approach, with high quality rated as A, medium quality as B, and low quality as C; very low quality of evidence was not considered. For evaluation of the quality of evidence according to GRADE [10], the following features were considered: study design, risk of bias, inconsistency, indirectness, imprecision, number of patients, effect, importance, and publication bias. The strength of recommendation was rated as 1 (strong) or 2 (weak).

The Delphi method was applied to arrive at a group opinion during the consensus conference.

Complete information, including the list of consensus conference workgroup domains (and topics noted below), and process regarding consensus conference participant selection, development and refinement of consensus statements, and modified Delphi methodology, including consensus polling, are previously reported in beforehand the in-person conference held in Prague, Czech Republic, Nov 13–15, 2022 [8].

## Results

### Heart Results

#### PICO 1: Heart (4 Statements)

In heart transplantation, for which heart should machine perfusion be performed?

• 1. The machine perfusion technique is safe (non-inferior) for heart preservation in transplantation.

Quality of Evidence: [moderate] Recommendation strength: [strong for].

The original statement proposed was: The machine perfusion technique is safe and effective for heart preservation in transplantation.” but reached a low quality of evidence and recommendation strength. The statement was rewritten based on the fact that even if same retrospective data show optimal organ preservation and clinical results [11, 12], randomized trials obtained non-inferior results [13, 14] and metanalysis were too heterogeneous (DBD and DCD together) to add meaningful data [15]. So, the new statement was changed highlighting non-inferiority, and the recommendation strength was increased from moderate to strong.

• 2. The use of machine perfusion reduces the cold ischemic time and, therefore, offers the possibility to prolong preservation time.

Quality of Evidence: [moderate] Recommendation strength [strong for].

The employment of MP limits the ischemic time to the time necessary for graft procurement, device instrumentation and heart transplantation independently by the transportation time that in this way can safely exceed the 4 h. Some reports describe very long support >16-17 h [16]. Recent data in DCD organ donation suggests further safe extension of the ischemic time in a wide variety of clinical settings [17].

• 3.1. Machine perfusion is a valuable tool in DBD to re-evaluate organ viability before implantation.

Quality of Evidence: [moderate] Recommendation strength: [strong for].

Lactates analysis permits to access organs during transportation, coronary angiography is possible when the heart is placed in the MP.

• 3.2. Machine perfusion is a valuable tool in DCD to assess and re-evaluate organ viability before implantation.

Whether normothermic regional perfusion is not feasible or available due to ethical and legal constraints, MP is the only possibility to assess DCD organs. DCD programs when a MP is employed permitted to obtain non inferior results compared to DBD programs [18].

Quality of Evidence: [moderate] Recommendation strength: [strong for].

• 4. Other devices for advanced graft preservation are under clinical investigation to extend the safe ischemic time.

Quality of Evidence: [low] Recommendation strength: [strong for].

The Guardian Registry showed valuable data about PGD reduction when controlled hypothermia is used for graft transportation compared with standard icebox [19, 20] also in extended donors [21].

#### PICO 2: Heart (1 Statement)

Heart In heart transplantation, which protocol/perfusate/perfusion strategy for *ex-vivo*/*ex-situ* heart perfusion leads to the best clinical outcomes post-transplant?

• 5.1. The current machine perfusion protocol(s) have been validated for clinical use in adult recipients.

Quality of Evidence: [moderate] Recommendation strength: [strong for].

In heart transplantation the availability of different protocols and perfusion strategies has been reduced by the presence of a single device for warm ESHP commercially available. The need of a standardization of the protocols of this commercially available MP has limited the possibility to have multiple protocols so there is a strong recommendation strength to strictly adhere to the unique methods utilized for all the trials on OCS.

• 5.2. The current machine perfusion protocols are feasible for clinical use in pediatric recipients.

Quality of Evidence: [moderate] Recommendation strength: [strong for].

No sufficient data regarding the use in pediatric recipients, however, the actual devices are recommended for donors >15 kg. In adult recipients suffering from end stage biventricular and univentricular congenital heart defects (CHD) machine perfusion is non-inferior compared to adult non-CHD patients [22].

#### PICO 3: Heart (3 Statements)

In heart transplantation, which biomarker/parameter is capable to predict the graft survival, graft function, primary non-function during *ex vivo* heart perfusion?

• 6. Angiography is a possible tool to assess coronary arteries of the heart during machine perfusion.

Quality of Evidence: [low] Recommendation strength: [strong for].

Angiography during MP is anecdotal and may be useful to evaluate anatomy more than quality. When concerns emerge during perfusion may be considered to rule-out organs with hidden coronary damages [12].

• 7. Lactate is the most commonly used parameter to assess the heart preservation during machine perfusion.

Although data from leading institutions [23] show that lactate levels doesn’t correlate with outcome the use is suggested by the consolidate use of the only warm ESHP commercially available. Data on DCD [24] seem to lower the importance of lactate in DCD donors.

Quality of Evidence: [low] Recommendation strength: [strong for].

• 8. Other biological/functional tools have to be developed to assess heart quality during machine perfusion

Quality of Evidence: [low] Recommendation strength: [strong for].

Although based on a single paper [25, 26] on current and future biomarkers the availability of new biomarkers to better evaluate the organ quality appears a possible gamechanger of the future of the technology thus improving the quality of the prediction of organ function and reducing the risk for PGD.

#### PICO 4: Heart (2 Statements)

In heart transplantation, which recipients will benefit from a heart assessed by machine perfusion?

• 9. The use of Machine perfusion is non-inferior to perform heart transplantation in VAD patients.

LVAD patients may be a surgical challenge and appear patients in which the MP technology may warrant superior outcomes permitting the surgeon to work without the hurry [26] in an elective setting. Many small retrospective reports support the safety of MP in this setting [27–29] but there is still a lack for well-designed trials in this setting,

Quality of Evidence: [moderate] Recommendation strength: [weak for].

• 10. Currently, there is consensus on recipient criteria that might indicate the need to perform machine perfusion

Quality of Evidence: [very low] Recommendation strength: [strong for].

The weight of the recipient’s features in Heart transplant appears a crucial factor for choosing the right way to preserve the donor graft. However, few small retrospective studies supported the use of MP in selected high-risk recipients as LVAD and CHD [30]. These patients however carry a high risk of mortality and ECMO support. Pediatric recipients might receive adult donor heart organs evaluated for transplantation in pediatric recipients. DCD donors over 15 kg are often preserved with ESHP [31]. The utilization of scores for selecting the right graft preservation strategy could represent a valuable attempt to justify the additional costs of MP in some healthcare systems with economic constraints.

### Lung Results

#### PICO 1: Lung (2 Statements)

**FIGURE 2:**
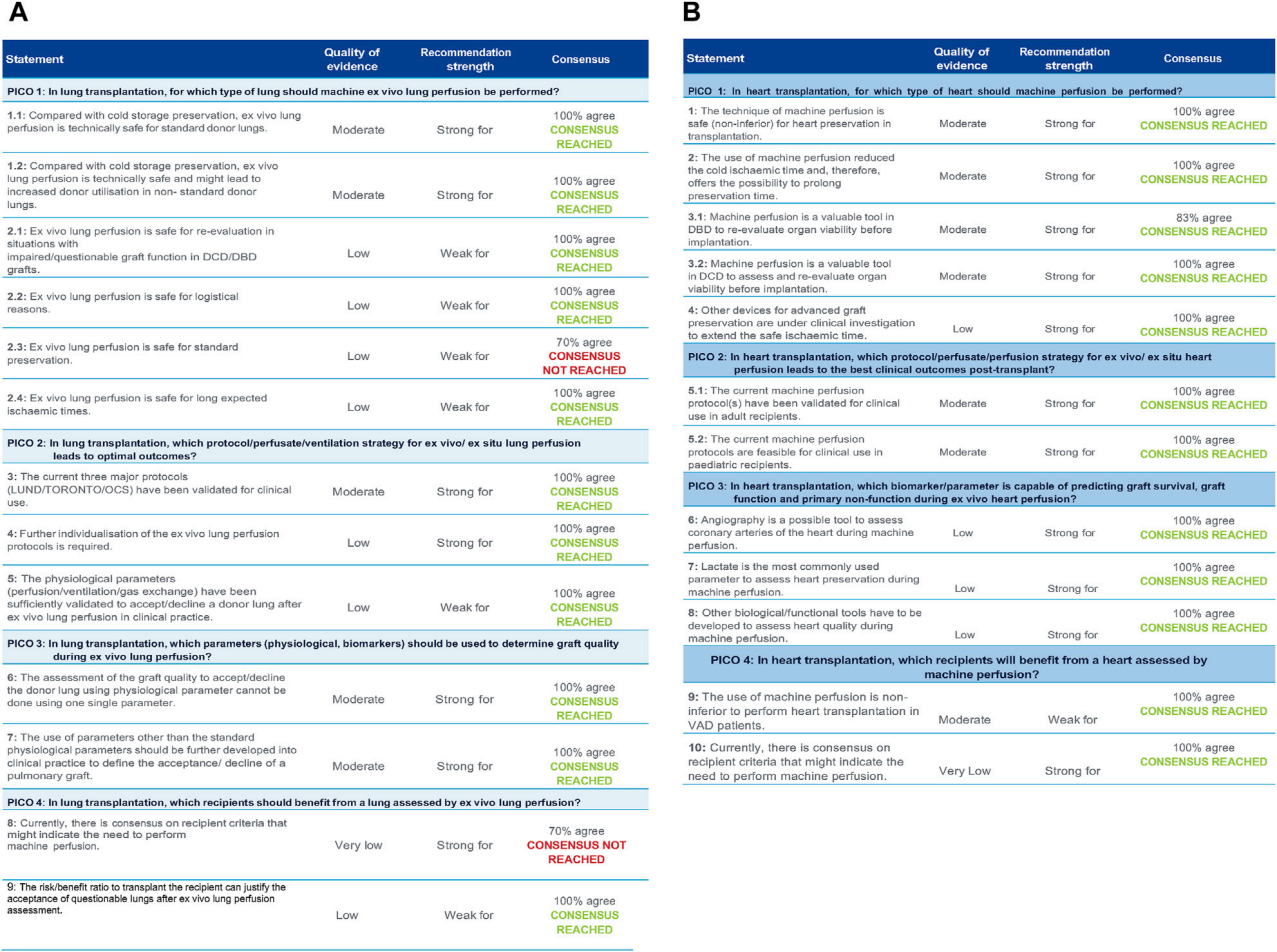
**(A)** Statements with quality of evidence, strength, and level of agreement during the Votation (heart) **(B)**. Statements with quality of evidence, strength, and level of agreement during the Votation (lung).

In lung transplantation, for which type of lung should *ex vivo* lung perfusion be performed? (Figure 2B)

• 1.1. Compared to cold storage preservation, *ex vivo* lung perfusion is technically safe for standard donor lungs.

Quality of Evidence: [moderate] Recommendation strength: [strong for].

Different clinical studies have investigated the use of EVLP for standard donor lungs [32–37]. The definition of standard vs nonstandard lung donors was strongly discussed since it appears a crucial limitation of the current literature since different manuscript tend to adopt different definitions [38–41]. The group agrees on the lack of robust data until now on the definition of the marginal or extended donors [42]. This definition should keep in consideration the differences between DBD, cDCD and uDCD. Also, based on local practices, not every DCD donor lung should be considered marginal or extended.

• 1.2. Compared to cold storage preservation, *ex vivo* lung perfusion is technically safe and might lead to increased donor utilization in non-standard donor lungs

Quality of Evidence: [moderate] Recommendation strength: [strong for].

Reported donor utilization rate after *ex vivo* lung perfusion from non-standard donor lungs ranges from 60%–90% based on case series and reported trials [34, 43–46]. The dynamic process of the quality of the organ during *ex vivo* lung perfusion may further complicate the definition of the advantage of MP to increase the donor utilization in non-standard donors. Recently, new evidence indicate also a paradigm shift in cold static storage preservation, where higher temperatures (avoiding freezing of the graft) are being investigated with promising results. The role of this new strategies for standard and non-standard donor lungs and the interaction with *ex vivo* lung perfusion should be investigated [47–50].

• 2.1. *Ex vivo* lung perfusion is safe for re-evaluation in situations with impaired/questionable graft function in DCD/DBD grafts.

Quality of Evidence: [low] Recommendation strength: [weak for].

Looking at the literature the heterogeneity of the reasons behind the use of MP [33, 34] in lung transplantation was debated and there was an agreement on analyzing separately the different indications for its usage. The recommendation strength behind the usage for reassessing the quality of the organ based on the current literature was considered low despite the clinical rationale that appears solid.

• 2.2. *Ex vivo* lung perfusion is safe for logistical reasons.

Quality of Evidence: [moderate] Recommendation strength: [strong for].

Currently standard use of *ex vivo* lung perfusion for logistical reasons is driven by local practices and clinical protocols and is based on the principle to prolong preservation times. The evidence for systematic use of *ex vivo* lung perfusion for extending preservation times is limited and needs further investigation. Some systems are portable and can be transported to the donor hospital. We have observed a tendency towards centralization of *ex vivo* lung perfusion which may impact the logistical use based on higher efficiency, reduced costs and centralization of expertise [51].

Also, new innovations in static cold preservation might need to redefine the role of *ex vivo* lung perfusion for logistical reasons alone.

• 2.3. *Ex vivo* lung perfusion is safe for standard preservation.

Quality of Evidence: [low] Recommendation strength: [weak for].

This statement didn’t reach the sufficient consensus (70%), further supporting the need for well-designed data in support of the use of MP in standard donors.

• 2.4. *Ex vivo* lung perfusion is safe for long expected ischemic times.

Quality of Evidence: [low] Recommendation strength: [weak for].

Based on the same discussion regarding logistical reasons for *ex vivo* lung perfusion, the clinical evidence to prolong ischemic times based on *ex vivo* lung perfusion is limited [52]. Further investigation to prolong the homeostasis of the graft is needed and experimental evidence is increasing to adjust the systems and protocols towards longer perfusion times [53]. Also, the combination of different intervals using *ex vivo* lung perfusion and static preservation strategies should be further investigated [50].

#### PICO 2: Lung (2 Statements)

In lung transplantation, which protocol/perfusate/ventilation strategy for *ex-vivo*/*ex-situ* lung perfusion leads to optimal outcomes?

• 3. The current 3 major protocols (LUND/TORONTO/OCS) have been validated for clinical use.

Quality of Evidence: [moderate] Recommendation strength: [strong for].

During the Consensus the 3 major protocols were described [33, 34, 54, 55], and the group agreed on the effectiveness of all of them to warrant optimal outcomes although no data could support the choice between each of them and direct comparisons are not possible.

• 4. Further individualization of the EVLP protocols is required.

Quality of Evidence: [low] Recommendation strength: [strong for].

The importance of cost-effectiveness studies to select the right preservation strategy based on clinical profile of donor and recipients was debated. The group agreed on the need of cost-effectiveness analysis to avoid the wasting of resources.

• 5: The physiological parameters (perfusion/ventilation/gas exchange) have been sufficiently validated to accept/decline a donor lung after *ex vivo* lung perfusion in clinical practice.

Quality of Evidence: [low] Recommendation strength: [weak for].

Although there is enough clinical data about the commonly accepted values of perfusion, ventilation and gas exchange parameters to decide whether an organ is usable or not after EVLP, the reality is that each group applies their own criteria, based on clinical practice, without robust evidence-based data to define the threshold to accept or reject a perfused graft [56].

#### PICO 3: Lung (2 Statements)

In lung transplantation, which parameters (physiological, biomarkers) should be used to determine graft quality during *ex vivo* lung perfusion?

• 6: The assessment of the graft quality to accept/decline the donor lung using physiological parameter cannot be done using one single parameter.

Quality of Evidence: [moderate] Recommendation strength: [strong for].

When evaluating the quality of a donor lung during *ex situ* lung perfusion, relying on a single physiological parameter is insufficient [57, 58]. Many different parameters and scores were presented during the session showing the potential room for moving from single parameters to multiparametric evaluations to discriminate the quality of the organ. Instead, a comprehensive assessment that considers multiple parameters (flow rate, compliance, gas exchange, airway pressures, lung weight) is essential to make informed decisions regarding the suitability of the lung for transplantation [59].

• 7: The use of parameters other than the standard physiological parameters should be further developed into clinical practice to define the acceptance/decline of a pulmonary graft.

Quality of Evidence: [moderate] Recommendation strength: [strong for].

There is a need for expanding beyond standard physiological parameters when assessing pulmonary grafts during *ex situ* lung perfusion. While traditional parameters like compliance, pulmonary vascular resistance (PVR), and oxygenation remain crucial, there’s a call to develop and incorporate additional parameters like biomarkers for inflammation or cellular damage [57, 58]. These novel indicators could enhance the accuracy of decisions regarding acceptance or rejection of donor lungs for transplantation [60]. The possibility to implement Machine-learning and AI technology was also highlighted as a future perspective.

#### PICO 4: Lung (2 Statements)

In lung transplantation, which recipients should benefit from a lung assessed by *ex vivo* lung perfusion?

• 8: Currently, there is consensus on recipient criteria that might indicate the need to perform machine perfusion.

It appears that the statement in question did not receive the required consensus of 70%. This reinforces the need for well-designed evidence to support the selection of recipients candidates for donors preserved with MP. The weight of the recipient’s features in Lung transplant still seems to be a challenging factor to consider.

• 9: The risk/benefit ratio to transplant of the recipient can justify the acceptance of questionable lungs after *ex vivo* lung perfusion assessment.

The discussion focused on the need to gather data to facilitate informed shared decision-making with patients to improve their experience and move towards person-centered care planning.

## Discussion

MP has been advocated as a tool to revolutionize the field of transplantation by:

• Increasing the number of organs,
• Improving the safety of the procedure,
• Reducing the burden of PGD,
• And converting an emergent procedure in a safe and calm elective procedure [55].

The technology has been separately developed for the heart and lung, with the lung as a trailblazer and a few groups in the world (Lund, Papworth, Toronto) as an upfront participant in clinical development. Given the possibility of assessing organ quality and widening the donor pool, the DCD has immediately become the natural clinical arena for growing the experience in the field until the possibility of reperfusing the organs in the donors through Normothermic Regional Perfusion has been envisioned [61].

CT MP has been developed as an alternative to the standard static-cold preservation method. The longer preservation of organs and real-time monitoring of organ quality may allow to redesign the allocation while also reducing or preventing ischemia-reperfusion injury. Ongoing improvements in MP protocols, particularly in extending the preservation duration, have opened up new possibilities for reconditioning and modifying diseased organs, as well as for tumor and infection therapies and regenerative approaches [62]. Lastly, the implementation of MP for in vivo-like preclinical studies that improve disease modeling has generated significant interest, creating an ideal interface for bioengineering and genetic manipulation [63]. In this perspective, large part of the innovation in the field of CT transplantation depends on how rapidly the research in this technology will evolve. Despite all these promises it is necessary to establish a methodological environment to warrant the use of this technology based on the unmet clinical needs of the patients and aimed at making the system economically sustainable in different healthcare systems.

## Heart

The change in the donor profile with the impressive increase of DCD [64] in many healthcare systems and the increase of mean donor age in Europe represents the first call for action to identify in which donors and in which recipients MP is necessary and when it may be helpful to warrant an improvement of patient’s outcomes. The PICOs of this consensus conference were designed to assess the heart and the lungs using the same methodology. Until the consensus, the only licensed system of MP for the heart was the OCS, with some upcoming data of the XVIVO coming from the first clinical application of this new technology [65]. PICO 2 and 3 for the heart were, therefore, mainly related to the protocol standardization coming from the OCS system.

The difference between DBD and DCD donors in terms of need of assessment and preservation was intensely discussed, and without envisioning the role of NRP [61] as an alternative for perfusing and evaluating organs, the MP was considered a valuable means to preserve and assess the donor hearts coming from DCD donors. The experience from all around the world with the NRP leave now opens the possibility of evaluating the heart with NRP and preserving the donors with SCS [66, 67]. During the discussion on the controversies around the utility of MP in the extended donors, one of the more controversial points was the demonstration of marginality for the extended donors and the demonstration of the reduction of the intrinsic risk (of PGD) carried from the donor. Scores like the Eurotransplant donor score [68] or the adapted Donor Risk score [69] have been advocated to demonstrate the complexity of the donor pool. Recently, the Donor Utilization Score [70] has shown differences between the European and US donor pools. Using a similar score to identify donors benefiting from preservation with MP could be a way to justify the additional costs carried by this technology. On the other hand, the authors shared the need to have well-designed RCTs or registries for LVAD recipients and CHD recipients to support the benefit of MP in this setting. After the impressive data coming from first XVIVO animal, experimental and clinical experiences [71–73], the next horizon will be to clarify the organs in which extending donor preservation (by Sherpapak or by XVIVO) may be sufficient to provide an improved outcome to the recipient and to which extent of extension the clinician may push the preservation time with each technology. Until now, the OCS has been the only technology that permits the assessment of the quality of the preservation and the intrinsic quality of the organ, and this retails a unique place to expand the donor pool.

The role of visual assessment is strongly dampened by the unloading of the heart, even if recently has been postulated a computerized system to assess the kinematics of *ex vivo* beating hearts undergoing normothermic perfusion on the TransMedics OCS [74]. This and similar tools may further fortify the possibility of the OCS to certify the quality of the graft.

The possible role of biomarkers [75] in this setting is another target for research to innovate the field of MP. The availability of a biomarker capable of appropriately predicting the hazard of PGD and delayed graft function may render the visual assessment unrelevant but also strengthen the advantage of dynamic strategies of perfusion over the impressive amount of data coming from the more reliable comparator that appears today, the Sherpapak.

One of the weaknesses of all the consensus was, in fact, the absence of a clear, unique comparator since icebox preservation has been poorly standardized and based on different cardioplegic solutions and delivery modalities (single shot, repeated before declamping, etc.).

The anecdotal demonstration that the *ex-vivo* preservation could mitigate the tissue damage that is expected after long ischemic times thus reverting the myocardial disarray is one of the most appealing issues supporting the possibility to expand donor pool through the implementation of MP [76].

In conclusion, MP appears the most attracting Innovation in a field that until now has been constraint by the lack of donors. MP has the possibility to exploit the number of CT transplants and redesign the field. Obviously, one of the variables in the pot is if the system will result sustainable and able to improve the outcomes of CT transplantation not only in terms of immediate outcomes but also during the mid and long-term thank to the possibility of modifying the immunogenicity of the grafts [77]. Having a certification of quality of the organ, the evolution from a center-based organization toward a national (or supranational in Europe) organization will be probably the natural evolution of the logistical and organizational pathways of CT transplantation permitting a broader allocation accounting also for HLA. The NOP in US and the Bridge in Sweden open the clear road from the center providing its own preservation strategy toward and Amazon-like organization where the organ may be evaluated at the arrival in the hospital before deciding to carry-on or not the operation for the single recipient identified with designed algorithms.

The recent perspective to prolong perfusion over 24 h [78] will further modify the pathways for organ allocation from the current standard toward a new model in which organ repair centers could also play a significant role.

## Lung

*Ex vivo* lung perfusion (EVLP) is a promising technology [56, 79]that allows donor lungs to be evaluated in a closed circuit outside of the body and extends lung donor assessment prior to final acceptance for transplantation. Compared to cold storage preservation, EVLP is technically safe for standard donor lungs and might lead to increased donor utilization in non-standard donor lungs. EVLP is also safe for re-evaluation in situations with impaired/questionable graft function in DCD/DBD grafts, logistical reasons, standard preservation, and long expected ischemic times. However, the evidence for the safety of EVLP for these situations is weak. The current three major protocols (LUND/TORONTO/OCS) have been sufficiently validated and have shown to be safe to accept/decline a donor lung after *ex vivo* lung perfusion in clinical practice. However, the assessment of the graft quality to accept or reject an organ should be performed in a holistic manner, taking into consideration different objective physiologic parameters (perfusion rate, vascular resistance, airway pressure, compliance, gas exchange, compliance, weight gain). Moreover, the use of parameters other than the standard physiological parameters (biomarkers) should be further developed into clinical practice to define the acceptance/decline of a pulmonary graft. Recent studies have shown that EVLP has diagnostic capabilities as an organ monitoring device and therapeutic potential to improve lung allograft quality when specific issues are encountered. An important aspect is the future development of EVLP as a reconditioning platform to translate and personalize different treatment strategies prior to transplantation.

The safety of EVLP for standard preservation statement did not reach a consensus. Despite clinical trials and retrospective studies have shown that recipients of EVLP-treated lungs have similar post-transplant survival rates compared to those who received conventionally preserved lungs, indicating that EVLP is non-detrimental in terms of mortality and retransplantation rates [80], they did not demonstrate superiority in standard donors, but it increases significant the costs and the optimal perfusion protocol and perfusate composition remain subjects of ongoing research. EVLP appears to be a safe and effective method for lung preservation, offering several advantages over traditional methods in selected cases, although further optimization and cost management are needed to fully realize its potential.

There is no absolute consensus on specific recipient criteria that indicate the need to perform EVLP. It is primarily employed to address the shortage of viable donor lungs by allowing the assessment, preservation, and reconditioning of marginal or high-risk donor lungs, which would otherwise be deemed unsuitable for transplantation.

It is important to note that EVLP is a relatively new technology, and its long-term effects are still being studied. Furthermore, the cost-effectiveness of EVLP compared to other methods of lung preservation is still being evaluated. Despite these limitations, due to ongoing improvements, EVLP has the potential to improve the quality and number of donor lungs available for transplantation, particularly through possible regenerative approaches to reprocessing and modifying originally marginal donor organs and in the use of DCD donors, but also in the future following the cardiac approach in the context of xenogeneic transplantation.

## Summary and Next Steps

The current evidence on MP is still weak, as stated in this document; however, there is a large consensus regarding the tremendous challenge that this technology offers to the expansion of the donor pool and to the reshaping of the logistics of CT transplantation. Facing the weaknesses of the current data, the group of experts agreed on the necessity of work in the direction of a European Registry for machine perfusion and DCD donation and on the need of cost-effectiveness studies to support the use of MP in CT transplantation.

## Data Availability

The original contributions presented in the study are included in the article/Supplementary Material, further inquiries can be directed to the corresponding author.

## Abbreviations

CET, Center of Evidence in Transplantation; CHD, Congenital heart defects; CT, Cardiothoracic; DBD, Donation after Brain Death; DCD, Donation after Circulatory Death; ECMO, Extra-Corporeal Membrane Oxygenation; ESOT, European Society of Organ Transplantation; EVLP, *Ex-vivo* Lung Perfusion; ICU, Intensive Care Unit; LVAD, Left Ventricular Assist Device; HT, Heart transplantation; MP, Machine perfusion; OCS, Organ Care System; PGD, Primary Graft Dysfunction; PICO, Population, Intervention, Comparator, and Outcome; RCTs, Randomized Controlled Trials; SCS, Static cold storage; uDCD, Uncontrolled Donation after Circulatory Death.
